# Optimized Purification of a Heterodimeric ABC Transporter in a Highly Stable Form Amenable to 2-D Crystallization

**DOI:** 10.1371/journal.pone.0019677

**Published:** 2011-05-13

**Authors:** Carmen Galián, Florence Manon, Manuela Dezi, Cristina Torres, Christine Ebel, Daniel Lévy, Jean-Michel Jault

**Affiliations:** 1 Université Joseph Fourier, Institut de Biologie Structurale, Grenoble, France; 2 UMR 5075 CNRS, Grenoble, France; 3 CEA Grenoble, Grenoble, France; 4 Institut Curie, Centre de Recherche, Paris, France; 5 CNRS, UMR-168, Paris, France; University of Cambridge, United Kingdom

## Abstract

Optimized protocols for achieving high-yield expression, purification and reconstitution of membrane proteins are required to study their structure and function. We previously reported high-level expression in *Escherichia coli* of active BmrC and BmrD proteins from *Bacillus subtilis*, previously named YheI and YheH. These proteins are half-transporters which belong to the ABC (ATP-Binding Cassette) superfamily and associate *in vivo* to form a functional transporter able to efflux drugs. In this report, high-yield purification and functional reconstitution were achieved for the heterodimer BmrC/BmrD. In contrast to other detergents more efficient for solubilizing the transporter, dodecyl-ß-D-maltoside (DDM) maintained it in a drug-sensitive and vanadate-sensitive ATPase-competent state after purification by affinity chromatography. High amounts of pure proteins were obtained which were shown either by analytical ultracentrifugation or gel filtration to form a monodisperse heterodimer in solution, which was notably stable for more than one month at 4°C. Functional reconstitution using different lipid compositions induced an 8-fold increase of the ATPase activity (*k*_cat_∼5 s^−1^). We further validated that the quality of the purified BmrC/BmrD heterodimer is suitable for structural analyses, as its reconstitution at high protein densities led to the formation of 2-D crystals. Electron microscopy of negatively stained crystals allowed the calculation of a projection map at 20 Å resolution revealing that BmrC/BmrD might assemble into oligomers in a lipidic environment.

## Introduction

Transport in and out of a cell or between its intracellular compartments is a vital process requiring many dedicated membrane proteins, and one of the largest families involved in these tasks is the ABC (ATP-binding cassette) transporters [1]. These transporters are ubiquitously distributed, from bacteria to man, and function by coupling ATP hydrolysis to vectorial transports, either import or export, of a wide diversity of substrates including amino acids, ions, sugars, complex organic molecules, peptides and even large proteins [2]. The basic architecture of an ABC transporter is four core domains with two hydrophilic nucleotide-binding domains (NBDs) that hydrolyze ATP to fuel the transporter and two transmembrane domains (TMDs) that allow the substrate to cross the membrane [3]. For importers, an additional subunit is involved in the capture and the delivery of the substrate to the TMDs [2]. The four core domains are either fused into a single polypeptide (full size transporters) or borne on separate polypeptides, from two to four. Transporters with one NBD genetically fused to one TMD are known as half transporters and work either as homodimers or heterodimers [4].

Some ABC members are involved in multidrug resistance (MDR) phenotypes due to their ability to efflux many structurally unrelated compounds [5]. Multidrug transporters represent a major threat to public health because they are responsible for cellular resistance to antibiotic, antifungal, antiparasitic and anticancer drugs in bacteria, yeast, protozoan parasites and human cancerous cells, respectively [5], [6], [7]. In humans, three ABC transporters are mainly involved in MDR phenotypes, namely the P-glycoprotein (Pgp or ABCB1), MRP1 (ABCC1) and BCRP (ABCG2) [8], [9]. Both Pgp and MRP1 are full size transporters while BCRP is a half transporter that self-associates to create a functional transporter [9]. Since the discovery of LmrA, a Pgp like half transporter in *Lactococcus lactis* [10], many bacterial ABC transporters have been assigned a putative MDR function based on bioinformatic classification [11]. New MDR bacterial ABC transporters have since been characterized at the molecular level [12], [13], [14], [15], [16]; notably the solving of the first high-resolution 3-D structure of a MDR ABC exporter [16], [17], suggesting that the minimal functional unit of these transporters (or related ones such as MsbA) is a homodimer. This is consistent with biochemical and other structural studies [17], [18], [19], [20], [21]. However, recent evidence has shown that some MDR bacterial ABC transporters function as heterodimers [22], [23], [24], [25], [26]. Interestingly, the presence of two different subunits allows such transporters, e.g. LmrC/LmrD, to work in an asymmetric mode regarding nucleotide binding and hydrolysis by their two NBDs [27]. This essential feature is shared with many eukaryotic ABC transporters including members of the human C family, such as MRP1 and CFTR, or the TAP1/TAP2 heterodimer [28], [29], [30]. To date, however, apart from this initial report on detergent solubilized LmrC/LmrD, there is a lack of information concerning the functioning mechanism of bacterial MDR ABC transporters that work as heterodimers.

We recently characterized a new heterodimeric ABC transporter from *Bacillus subtilis*, YheI/YheH [31], and given its significant homology to LmrC/LmrD of *L. lactis*, we hereafter call it BmrC/BmrD. Importantly, the overexpression of both subunits, BmrC and BmrD, were required to detect a multidrug transport activity in inside-out *E. coli* membrane vesicles. Moreover, expression of both *bmrC* and *bmrD* genes was strongly increased upon exposition of *B. subtilis* to many antibiotics, supporting a role for BmrC/BmrD as a new multidrug transporter [31]. Here, we have set up a purification protocol for this heterodimeric transporter allowing the recovery in high yield of an active, stable and monodisperse heterodimeric transporter in a detergent solubilized state. An optimized reconstitution protocol into proteoliposomes allowed this transporter to display a high ATPase activity, about 8-times higher than in detergent solution. Moreover, 2-D crystals of this transporter were obtained in membrane in an ADP/vanadate trapped conformation thus confirming the quality of the preparation. Negative staining of these 2D crystals allowed us to obtain a projection map at a resolution of 20 Å. This reveals a possible supramolecular organization of BmrC/BmrD heterodimers in a lipidic environment.

## Results

### Overexpression of BmrC/BmrD

Previously, we co-expressed BmrC and BmrD-His_6_ in *E. coli* BL21(DE3) thereby obtaining membrane vesicles highly enriched in these two proteins, and we showed that they were both required to detect a transport activity of several drugs [31]. Initial attempts to purify this heterodimer transporter from these vesicles led to some loss of the untagged subunit (i.e. BmrC) when Ni^2+^ affinity chromatography was performed in the presence of different detergents (see Figure S1). Thus, although the two subunits interact in the membrane, addition of a high concentration of detergent required to efficiently solubilize the transporter presumably weakens the interaction between them (or the association with stabilizing lipids). This led to a partial loss of the untagged subunit during the subsequent affinity chromatographic step. To overcome this hurdle, we decided to add an *N*-terminal hexahistidine tag to the BmrC subunit and thus to work with both subunits tagged. Higher amounts of His-tagged BmrC were obtained in the membrane preparations leading to an excess of this subunit as compared to the His-tagged BmrD (Fig. S1). Yet, no difference in substrate transport between the mono-tagged and the bi-tagged constructs was observed for membrane vesicles containing overexpressed BmrC/BmrD (data not shown). A typical preparation starting from 1 liter of culture medium yielded ∼150 mg of total membrane proteins, of which at least 50% corresponded to BmrC/BmrD (Fig. S1).

### Solubilization screen

**Figure 1:**
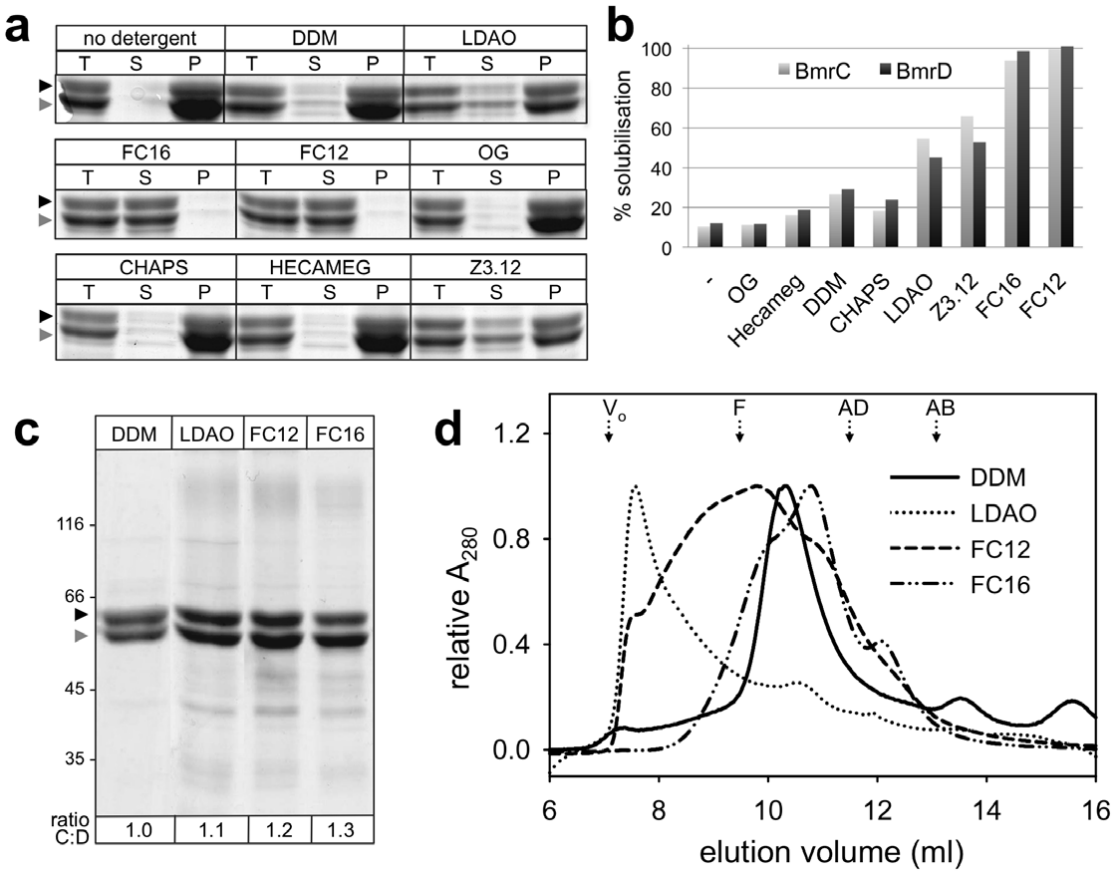
Purification of BmrC/BmrD using different detergents. (***a***) BmrC/BmrD solubilization by non-ionic and zwitterionic detergents was performed at 2 mg/ml protein and 10 mg/ml detergent and assessed by Coomassie Blue stained SDS-PAGE of the soluble (S) and insoluble (P) material obtained from 15 µg of total protein sample (T). Note that the strong densities observed in some of the pellet bands probably arise from difficulties encountered while resuspending the pellet that led to inhomogeneous samples. Black arrowhead, BmrD; grey arrowhead, BmrC. (***b***) The intensities of the bands corresponding to the S and T samples were quantitated using Image Gauge software (Fuji Film Science Lab) and the solubilization yields were calculated as the ratio of each protein in the supernatant (S) relative to its amount in the membrane fraction (T). (***c***, ***d***) Protein purified by nickel affinity chromatography in the presence of the indicated detergents was analyzed by (***c***) Coomassie Blue stained SDS-PAGE (5 µg per lane, except for DDM : 4 µg) and (***d***) size-exclusion chromatography performed on a Superdex 200 10/300 GL column using 0.05% (w/v) DDM in the elution buffer. Elution volumes of proteins used for column calibration are indicated above: ferritin (F, 440 kDa), aldolase (AD, 158 kDa), albumin (AB, 67 kDa), V_o_: void volume. The intensities of the BmrC and BmrD bands in the polyacrylamide gel in ***c*** were quantitated and the ratio BmrC∶BmrD (ratio C∶D) was indicated under each lane. Black arrowhead, BmrD; grey arrowhead, BmrC.

Membrane preparations highly enriched in BmrC/BmrD were incubated with a sampling of commonly used non-ionic and zwitterionic detergents. The non-ionic detergents tested were *n*-dodecyl-ß-D-maltoside (DDM) and its thio-derivative *n*-dodecyl-ß-D-thiomaltoside (DOTM), *n*-octyl-ß-D-glucoside (OG) and 6-O-(n-heptylcarbamoyl)-methyl-α-D-glucopyranoside (HECAMEG). As shown in Figure 1*a–b*, DDM performed best among the tested non-ionic detergents, although the yield was rather low with less than 30% of the protein being solubilized. Similar extraction yields were obtained with DOTM (not shown). In contrast, OG was unable to solubilize BmrC/BmrD. The zwitterionic detergents tested were lauryldimethylamine oxide (LDAO), n-dodeclyphosphocholine (FC-12), n-Hexadecylphosphocholine (FC-16), *n*-dodecyl-N,N-dimethyl-3-ammonio-1-propanesulfonate (Z3-12), and and 3-[(3-cholamidopropyl)-dimethylammonio]-1-propane sulfonate (CHAPS). With the exception of CHAPS, all solubilized BmrC/BmrD more efficiently than the non-ionic detergents. The two fos-cholines had by far the best extraction efficiencies, close to 100%. Additionally, a mixture of 0.1% DDM and 1% polyoxyethylene-8-dodecylether (C_12_E_8_) that was successfully used to solubilize Sav1866 [17], was also tried but did not give any improvement as compared to DDM alone (not shown). All mild detergents (i.e. with an extraction efficiency <40%) solubilized BmrD slightly better than BmrC (Fig. 1*b*). Given the excess of BmrC in the membrane fraction, this observation suggests that excess subunits that are not assembled into heterodimers are misfolded in the membrane and therefore less readily solubilized (see also the Discussion section). In contrast, LDAO and Z3.12 appeared to solubilize the BmrC subunit more efficiently than BmrD. This effect was even more pronounced using the stronger detergents (i.e. FC12 and FC16) that solubilized almost entirely both proteins. This second category of detergents thus maintained an excess of BmrC subunit in the solubilized fraction.

### Purification of BmrC/BmrD using four selected detergents

On the basis of their high efficiency in the solubilization screen, FC12 and FC16 were further retained for purification of BmrC/BmrD. In addition, despite their low extraction efficiency, LDAO and DDM were also included in our purification trials because they have been successfully used for purifying and characterizing other ABC transporters [32], [33], and are among the preferred detergents for crystallization of membrane proteins [34]. Membrane solubilization and Ni^2+^-chelate affinity chromatography were carried out with each of these four detergents. As expected, protein yield correlated with extraction efficiency. Fos-cholines allowed purifying 40–50 mg of protein per liter of culture medium, while protein yields were about ten times lower when DDM or LDAO were used. Analysis by sodium dodecyl sulfate polyacrylamide gel electrophoresis (SDS-PAGE) reveals that, for each detergent, the proteins were recovered with a similar degree of purity (Fig. 1*c*). However, the apparent stoichiometry of the purified BmrC and BmrD subunits seems to be dependent on the detergent used. Although the intensity of the Coomassie-stained bands is an approximation of the actual amount of protein, an apparent excess of BmrC was observed in the fos-choline purified samples (cf. Fig. 1*c*). Thus, DDM was the only detergent that allowed the purification of both subunits in an apparent equimolar complex.

To further characterize the purified complex, it was loaded onto a Superdex 200 10/300 GL column, previously equilibrated with 0.05% (w/v) DDM in the mobile phase. This condition was reported not to rescue aggregation of a protein initially solubilized by a different detergent [35]. As shown in Figure 1*d*, purification of the complex in FC12 or LDAO yields a polydisperse and substantially aggregated protein. In contrast, BmrC/BmrD purified in FC16 or DDM is not aggregated, with only DDM achieving a good monodispersity of the complex, with a major peak that elutes at ∼10.5 ml. In agreement with these observations, measurements of the ATPase activity of the complex purified in different detergents showed that only DDM-purified BmrC/BmrD was able to hydrolyse ATP to a significant level. The three other detergents led to an ATPase activity of at most 12% of that obtained with the DDM preparation (Fig. S2). In addition, when BmrC/BmrD purified in the presence of FC16 or LDAO was used to grow 2-D crystals (see below), only protein aggregates were observed by electron microscopy in the preparation.

Thus, based on the apparent homogeneity and high ATPase activity, DDM was chosen as the detergent for BmrC/BmrD purification. Further optimization of the purification protocol consisted in shortening the induction time for protein production from 16 to 6 hours. This led to an increase of solubilization efficiency up to 50% (not shown) and thus higher protein yields. Presumably, the 16 hours induction time resulted in a higher amount of misfolded transporter in the membrane. This misfolded protein was refractory to DDM solubilization. Using a 6 h. induction time, a typical preparation from 1 liter of culture medium yielded ∼12 mg of purified BmrC/BmrD in detergent solution, with an ATPase activity of 231±17 nmol·min^−1^·mg^−1^ (0.56±0.04 s^−1^; n = 5 measurements with two different purification batches).

The ATPase activity of the individual BmrC and BmrD subunits were also analyzed separately after purification in DDM. However, although membrane expression levels of the separate BmrC and BmrD proteins were similar to that obtained when they were jointly overexpressed [31], the solubilization step was found to be quite inefficient for the isolated subunits and affinity chromatography separations resulted in highly contaminated proteins, especially for the BmrD preparation (Fig. S3 *a*). This result suggests that, in the absence of its twin subunit, a single subunit is unable to associate into a stable homodimer and likely folds improperly in the membrane. Compared with BmrC/BmrD purified in the same manner, a much lower level of ATPase activity was obtained for fractions containing BmrC or BmrD alone (∼80 and ∼60 nmol·min^−1^·mg^−1^, respectively; Fig. S3 *b*) that were not sensitive to vanadate inhibition. As a control, a double mutant where the invariant Lys residue in the Walker-A motif of each subunit was mutated to an Ala residue (BmrC K377A/BmrD K469A) exhibited also a low residual activity insensitive to vanadate inhibition (Fig. S3 *b*). Presumably, the vanadate-insensitive ATPase activity in these different fractions is due to some contaminants bearing an ATPase activity.

### Characterization of DDM-purified BmrC/BmrD

**Figure 2:**
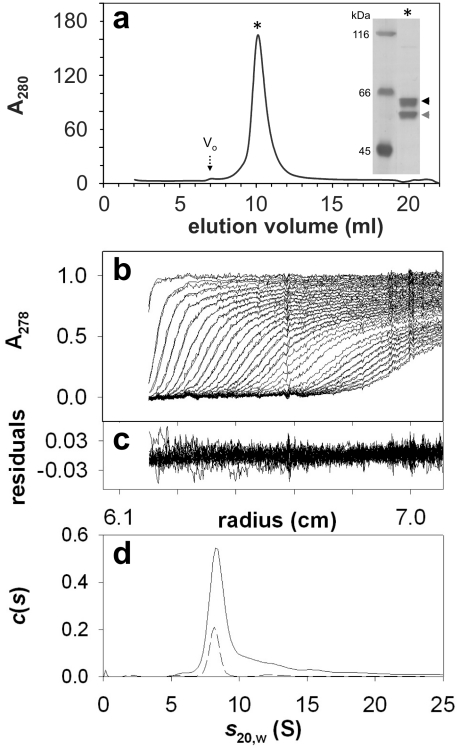
DDM-purified BmrC/BmrD is monodisperse. (***a***) Size-exclusion chromatography of DDM-purified protein (∼0.3 mg) was performed as indicated in fig. 1*d*. Inset: Silver-stained SDS-PAGE of protein eluted at 10 ml. Black arrowhead, BmrD; grey arrowhead, BmrC. V_o_: void volume. (***b–d***) Samples of nickel-affinity purified BmrC/BmrD (0.85 mg/ml) kept frozen at −80°C after flash freezing in liquid N_2_, were thawed and analyzed by sedimentation velocity measurements at 20°C and 42000 revolutions per minutes. (***b***) Superposition of selected sedimentation profiles obtained at 278 nm and the corresponding fitted curves using continuous size distribution analysis. (***c***) Superposition of the residuals. (***d***) *c*(*s*) curves for the stock protein (continuous line) and the protein diluted 4 times in dialysis buffer (dashed line).

Analysis by size-exclusion chromatography (SEC) of the whole complex purified after a 6 hours induction shows an elution profile with a more symmetrical peak (cf. Fig. 2*a*). This sharper, Gaussian-like peak, reveals that the reduction of the induction time not only increased the purification yield, but also improved the homogeneity of the purified proteins [36]. Silver staining of the material eluted in this peak shows that the BmrC/BmrD complex was relatively pure as low molecular weight contaminants were removed and only a very faint contaminant remained with an apparent molecular mass of ∼100 kDa. Comparison of the elution volume of the major peak with that of protein standards of known size allows an estimation of the molecular mass of the BmrC/BmrD detergent complex of ∼310 kDa (more precisely the Stokes' radius *R*_S_ is ∼6.1 nm). This mass is about twice the protein part of a BmrC/BmrD/detergent complex, given that the theoretical molecular masses of 6×His-tagged BmrC and BmrD proteins are 67 and 77 kDa, respectively. A comparable molecular mass (300±30 kDa) was obtained by this approach for the homodimeric BmrA transporter (66.2 kDa for the monomer), which was indeed shown to be a homodimer in a DDM solution using analytical ultracentrifugation [21].

To precisely assess the oligomeric state of BmrC/BmrD in DDM solution, analytical ultracentrifugation experiments were also performed. Data from a typical sedimentation velocity experiment are shown in Figure 2*b–d*. The experimental points were fitted in terms of distribution of non-interacting particles and the goodness of the fit was checked by the low values of the residuals and their random distribution. The majority of the protein sediments at *s*_20,w_ = 8.6 S (*s*_exp_ = 5.8 S), a value close to that measured for the dimeric BmrA (*s*_20,w_ = 8.9±0.3 S [21]). Measurements with interference optics (not shown) led to an estimate of the amount of bound detergent plus lipids of 0.5 g/g (compared to 1.5 g/g estimated for BmrA). Such a complex of BmrC/BmrD/DDM (neglecting the possible lipids associated), if slightly elongated or globular (frictional ratio of 1.5-1.25, corresponding to a hydrodynamic radius *R*_H_ of 6.1-5.0 nm) would sediment at *s*_20,w_ values of 7.2–8.7 S. On the other hand, each of the isolated subunits would have *s*_20,w_ values of 4.6–5.5S, and a dimer of heterodimers would have *s*_20,w_ values of 11–14 S. From these calculations, and the comparison with BmrA, it is clear that the main species in DDM solution is the BmrC/BmrD heterodimer. Some larger species were also detected at higher *s_20,w_* values (see Fig. 2*d*) consistent with the tendency of the heterodimer to oligomerize (see below).

**Figure 3:**
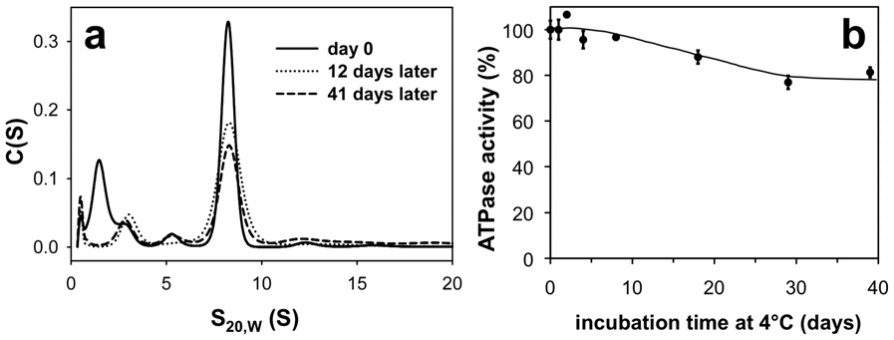
Stability of DDM-purified BmrC/BmrD. (***a***) After nickel-affinity and size-exclusion chromatography in the presence of DDM, purified BmrC/BmrD protein at 0.11 mg/ml was immediately subjected (day 0) to sedimentation velocity measurements or incubated at 4°C for 12 or 41 days before performing analytical ultracentrifugation experiments at 42000 rpm and 20°C. The superposition of the *c*(*s*) curves from data obtained at 278 nm at different times is shown. (***b***) ATPase activity of nickel-affinity purified BmrC/BmrD was determined in 0.05% (w/v) DDM after incubation at 4°C for the indicated times. The values are expressed as the mean ± SD for three measurements, relative to the ATPase activity obtained without prior incubation at 4°C (244±10 nmol·min^−1^·mg^−1^).

Having shown that BmrC/BmrD were essentially monodisperse in DDM and that they behaved as a heterodimer, we next studied the stability of this complex during a long-term storage at 4°C. This parameter has been empirically defined as one of the key factors for successful crystallization of membrane proteins [36]. Sedimentation velocity experiments were therefore performed directly after the size exclusion chromatography. They show a high level of homogeneity of purified BmrC/BmrD, which was essentially maintained after storing the protein 41 days at 4°C. More than 70% of the protein was recovered (Figure 3*a*), with the remaining being possibly stuck on the glassware. The effect of storage at 4°C on the ATPase activity of BmrC/BmrD was also monitored in a separate experiment. As shown in Figure 3*b*, ∼80% of the initial ATPase activity was conserved after 39 days of storage.

### ATPase activity of BmrC/BmrD in DDM is sensitive to vanadate and stimulated by Hoechst

**Figure 4:**
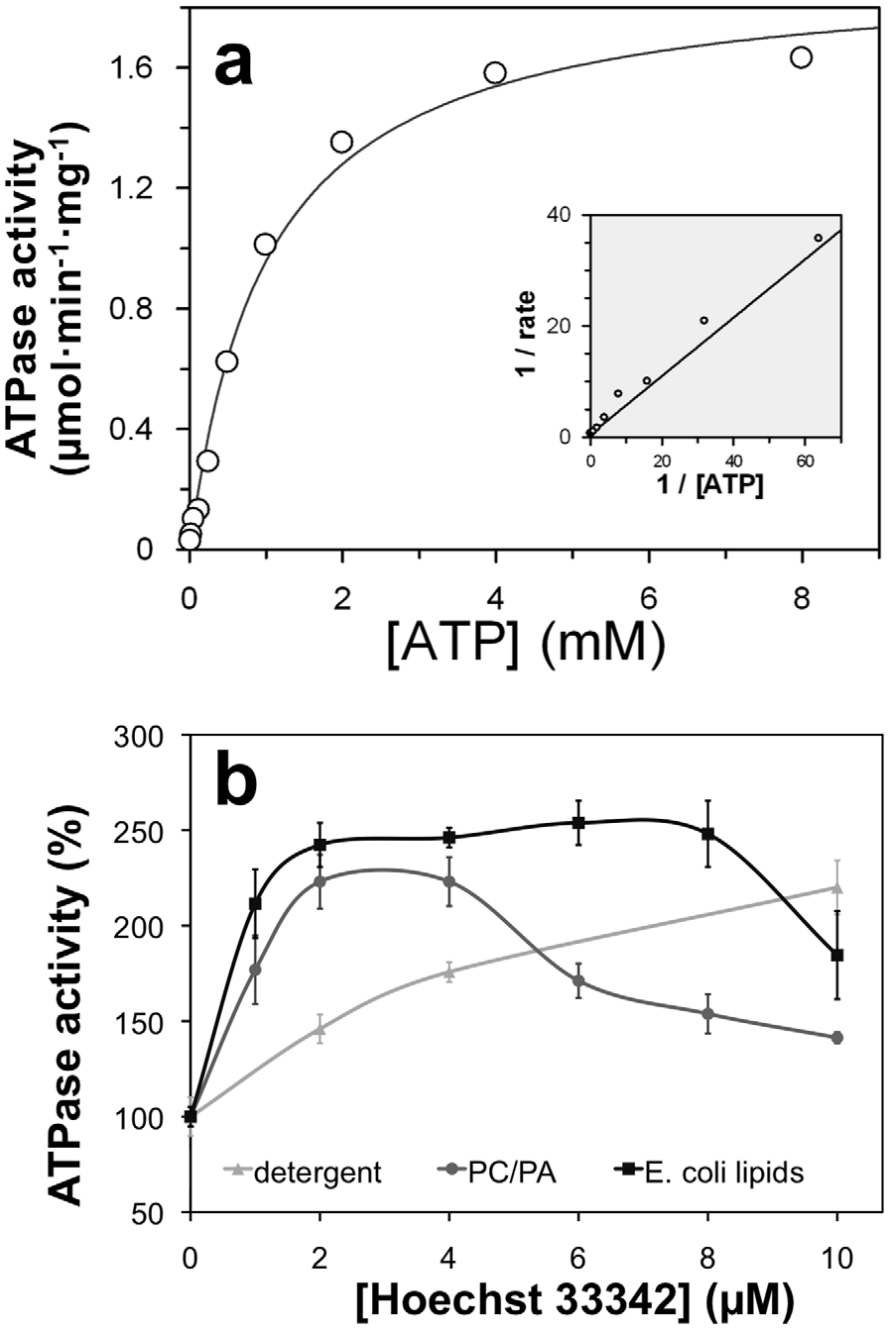
Reconstitution of BmrC/BmrD into proteoliposomes. BmrC/BmrD were reconstituted at lipid to protein ratio of 40 (w/w) by detergent removal from different solubilized mixtures of DDM/lipid/protein. (***a***) ATPase activity of proteoliposomes was assessed at 4 mM ATP. *E. coli*: total extract of *E. coli* phospholipids; PC: egg-phosphatidylcholine; PA: egg-phosphatidic acid; chol: cholesterol; DOPC: dioleoylphosphatidylcholine; DOPG: dioleoylphosphatidylglycerol; CL: cardiolipin. Lipid composition was as follows (w/w): PC/PA 9∶1; PC/PA/chol 7∶1∶2; DOPC/DOPG 9∶1; PC/CL 8∶2. (***b***) Cryo-electron microscopy of reconstituted proteoliposomes with EPC/EPA. All proteoliposomes were unilamellar and showed a continuous lipid bilayer. Scale bar: 50 nm. (***c***) ATPase activity measured after reconstitution of BmrC/BmrD with EPC/EPA or *E. coli* lipids at lipid to protein ratio of 40 (w/w) and 100 (w/w). (***d***) Protein incorporation in proteoliposomes after fractionation in a sucrose gradient. The amount of BmrC/BmrD in every fraction was measured by quantitation of the protein bands revealed by immunoblot (the intensity of both bands were added) and normalization to the total amount of BmrC/BmrD loaded onto the sucrose gradient (cf. Materials and Methods). Most proteins were reconstituted in proteoliposomes running at 2.5%–5% sucrose without significant aggregates at 30% sucrose.

We have previously shown that in *E. coli* membranes, the ATPase activity of BmrC/BmrD was sensitive to vanadate inhibition [31], and consistent with our previous result, 81±2% of the ATPase activity of DDM solubilized BmrC/BmrD was inhibited by 0.5 mM vanadate. As Hoechst 33342 was previously found to be a substrate efficiently transported by BmrC/BmrD [31], its effect on the ATPase activity on purified BmrC/BmrD was studied. Addition of increasing concentrations of Hoechst stimulated ATP hydrolysis by BmrC/BmrD about two-fold when the Hoechst concentration reached ∼10 µM (cf. Fig. 4*b*, light gray trace). These results reveal that not only the ATP-binding sites of BmrC/BmrD were kept intact throughout the purification protocol, but also that the drug-binding site as well as the conformational coupling between both sub-domains are functional in the DDM purified transporter. Other substrates shown previously to be transported by BmrC/BmrD, namely mitoxantrone, doxorubicin and BCECF [31], or capable to inhibit the Hoechst transport such as vinblastine were also tested but they resulted in a modest stimulation of about 20% of the ATPase activity of BmrC/BmrD (not shown).

### Reconstitution of BmrC/BmrD into proteoliposomes

**Figure 5:**
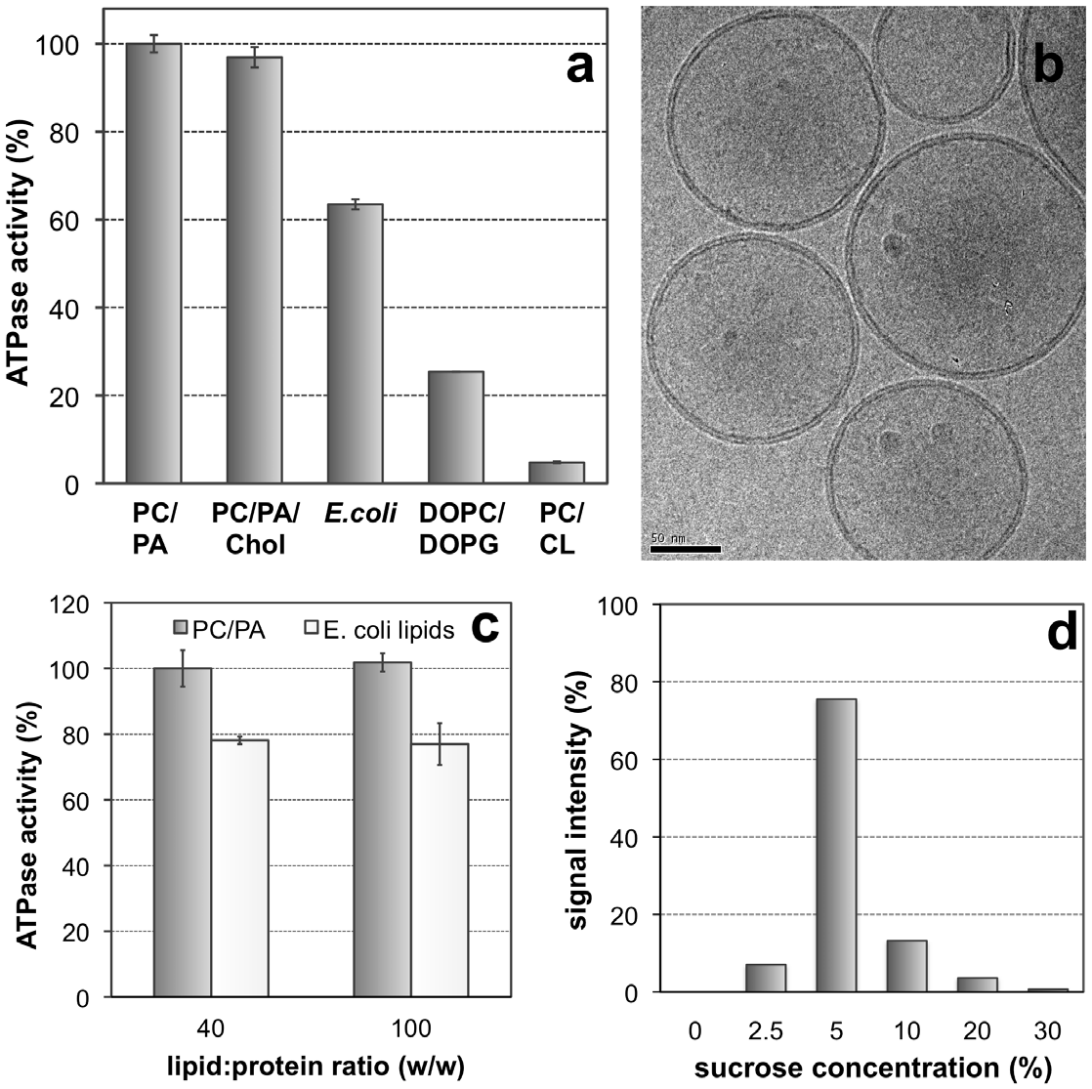
Reconstituted BmrC/BmrD displays a high and substrate-stimulated ATPase activity. (***a***) BmrC/BmrD was reconstituted into proteoliposomes using a mixture of PC/PA (9∶1, molar ratio), and the effect of ATP/Mg^2+^ concentration on the initial ATPase velocities was determined. The solid line represents the best fit of the data to Michaelis-Menten equation (using the GraFit 5 software from *Erithacus*), allowing the determination of V_max_ = 1.93±0.08 µmol·min^−1^·mg^−1^ (k_cat_∼4.7 sec^−1^) and *K*_m_ = 1.0±0.1 mM. Inset shows the Lineweaver-Burk Plot of the data. (***b***) The ATPase activity of BmrC/BmrD reconstituted in proteoliposomes, prepared either with PC/PA (black) or *E. coli* lipids (dark gray), was studied in the presence of increasing concentrations of Hoechst 33342. For comparison, the ATPase activity of BmrC/BmrD in 0.05% (w/v) DDM is shown (light gray). The values are expressed as the mean ± SD for three measurements, relative to the ATPase activity obtained without addition of Hoechst 33342.

BmrC/BmrD was reconstituted into proteoliposomes after detergent removal by hydrophobic adsorption onto polystyrene beads (Biobeads) starting from a mixture of lipid/protein/DDM. This procedure has invariably led to the reconstitution of different membrane proteins in unilamellar proteoliposomes, homogeneous in size, with a low ionic permeability and total protein incorporation (reviewed in [32], [37], [38]). Different lipid mixtures were tested including egg-phosphatidylcholine (PC)/egg-phosphatidic acid (PA), PC/PA/cholesterol (Chol), *E. coli* Lipids, dioleoylphosphatidylcholine (DOPC)/dioleoylphosphatidylglycerol (DOPG) and DOCP/cardiolipin (CL), and ATPase activities of the resulting proteoliposomes were measured. In all cases, ATPase activity drastically increased, up to 8-fold compared to the protein in detergent. The highest ATPase activity, ∼2 µmol/min/mg protein, was attained when reconstitution was performed with PC and PA (9∶1 molar ratio; Fig. 5*a*). Although chemically similar, the mixture DOPC/DOPG resulted in much lower ATPase activity. When reconstitution was performed with *E. coli* lipids (total polar extract), the ATPase activity of BmrC/BmrD was ∼30% lower (n = 5). Finally, addition of cardiolipin to the reconstitution mixture was detrimental to BmrC/BmrD ATPase activity. Proteoliposomes were then analyzed by cryo-electron microscopy. As expected, they showed a homogeneous population of unilamellar vesicles of 100–150 nm diameter. Importantly, the lipid bilayers were continuous. No deformed or open vesicles were found that would be associated with an increased passive permeability (Fig. 5*b*). The incorporation and protein repartition among the population of vesicles were evaluated by two additional experiments. First, BmrC/BmrD were reconstituted at different lipid/protein ratio and specific ATPase activities were measured. At low protein densities (lipid/protein molar ratio of 40 and 100), the ATPase activities observed were similar (Fig. 5*c*). Next, proteoliposomes were submitted to discontinuous flotation gradients, as previously described [37], with successive layers containing 30, 20, 10, 5 and 2.5% sucrose. The fractions were collected and analyzed for protein contents by immunoblot using anti-His HisProbe-HRP revealing that most of the BmrC/BmrD were found at 2.5%–5% interface. This corresponded to the expected densities for proteoliposomes (Fig. 5*d*). Moreover, a very faint signal was found at 30% sucrose demonstrating that a negligible amount of proteins aggregates during reconstitution.

We also characterized the morphology and ATPase activity of proteoliposomes obtained after detergent removal from a PC/PA mixture solubilized in different detergents. Indeed, lipid/detergent intermediates formed during detergent removal are specific for the detergent, so that the morphology of the resulting liposomes depends on the detergent used (for review see [38]). The highest ATPase activity (∼2 µmol·min^−1^·mg^−1^) was measured when proteoliposomes were prepared with DM (Fig. S4). No significant differences were obtained between DM and DDM mediated reconstitutions, the ATPase activities of the resulting proteoliposomes were 1.9±0.4 (n = 5) and 1.7±0.6 µmol·min^−1^·mg^−1^ (n = 10), respectively. In contrast to previous reports on other ABC transporters [32], reconstitution using Triton X-100 was less favorable for BmrC/BmrD ATPase activity than the maltoside-derivatives (Fig. S4). Finally, reconstitution with OTG gave essentially multilamellar proteoliposomes resulting in a low ATPase activity (Fig. S4).

Direct incorporations into preformed liposomes destabilized with a sub-solubilizing concentration of DDM were also performed. This reconstitution method has been shown to ensure a unique orientation of proteins into liposomes with large extramembranous domains of membrane proteins facing the extracellular medium [37], [38]. Here, this led to a ∼20% increased of the ATPase activity as compared to incorporation from a fully solubilized mixture of lipid/proteins/detergent. Thus we can estimate that most of the proteins (>80%) was incorporated in the right orientation (i.e. inside-out topology) competent for ATP hydrolysis when reconstituted from a fully solubilized mixture of lipid/proteins/detergent.

Because the reconstitution protocol using fully solubilized lipids yielded a high and consistent ATPase activity, we chose this technique to determine the kinetic constants for ATP hydrolysis by BmrC/BmrD incorporated into proteoliposomes prepared from PC/PA solubilized by DDM (Fig. 4*a*). Data obtained using increasing ATP concentrations were fitted to the Michaelis-Menten equation with a calculated *V*_max_ of 1.93±0.08 µmol·min^−1^·mg^−1^ (k_cat_∼4.7 s^−1^) and a *K*_m_ value of 1.0±0.1 mM for ATP. Like the DDM-solubilized transporter, the ATPase activity of the reconstituted BmrC/BmrD was stimulated upon Hoechst addition, irrespective of the lipids used during the reconstitution (Fig. 4*b*). Our data show that the ATPase activity of BmrC/BmrD can be increased 8 times upon reconstitution into proteoliposomes.

### 2-D crystallization of BmrC/BmrD

**Figure 6:**
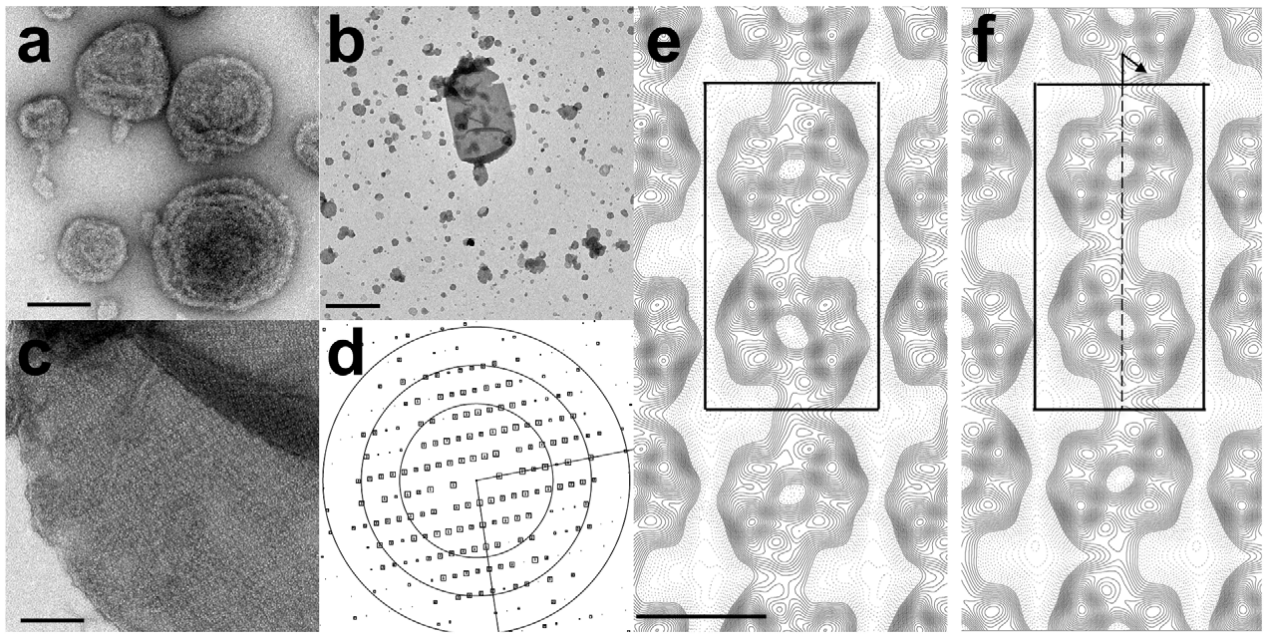
2-D crystallization of BmrC/BmrD. BmrC/BmrD was reconstituted with PC/PA/cholesterol lipid mixture at 0.6 (w/w) as described in the Materials and Methods section in the absence (***a***) or in the presence (***b***, ***c***) of ATP/MgCl_2_/vanadate. (***b***, ***c***) Electron micrographs of negatively stained 2-D crystals. (***d***) Representation of amplitudes of Fourier components calculated for one image of a negatively stained crystal. Numbers and box sizes correspond to the spot IQ value, with spots of the highest signal-to-noise ratio having an IQ of 1 and the lowest of 5 [84]. The three black circles are at radii corresponding to 1/35, 1/24, 1/18 Å^−1^. (***e***, ***f*** ) Projection maps of BmrC/BmrD at 21 Å resolution calculated from merged amplitudes and phases from five independent crystals with p1 symmetry and p12_1_-b symmetry, respectively. Solid lines indicate density above the mean. Negative contours are indicated by dotted lines. The lattice parameters are a = 122 Å, b = 236 Å and gamma = 90°. The unit cell, dark box, contains two supramolecular entities (one being outlined by a dotted, light gray box in ***e***), related by a screw axis along b (dotted arrow in ***f*** ). Scale bars are equal to 100 nm (***a*** and ***c***), 1 µm (***b***).

2-D crystals were obtained after addition of lipids at low lipid/protein ratio 0.5–1 (w/w) followed by slow detergent removal using Bio-Beads. Vesicles homogeneous in size, 100–300 nm diameter, and containing densely packed proteins were obtained with several lipid mixtures including e.g. dimyristoylphosphatidylcholine, DOPC, PC/PA (9/1, w/w), and PC/PA/cholesterol or PC/PA/cardiolipin (7/2/1, w/w/w). In the absence of ATP/MgCl_2_, 2D crystals were never obtained although proteins were present at high densities in the membrane, as seen by negative staining. Strikingly in all cases, irrespective of the lipids used, membranes of proteoliposomes were not flat as usually observed in 2D crystallization trials but showed several deformations or ripples (Fig. 6*a*). It is worth mentioning that similar deformations have been observed after reconstitutions of MsbA in the absence of ATP/MgCl_2_ (D. L., unpublished data). However, when reconstitutions were performed in the presence of ATP/MgCl_2_/vanadate, deformation of the membrane was not observed and several vesicles contained proteins packed in 2-D arrays. The size of the crystalline patches increased with the duration of reconstitution and the largest 2D crystals were obtained after 3 days of reconstitution. The best conditions were found after reconstitution in the presence of PC/PA or PC/PA/cholesterol lipid mixtures, leading to large tubular shaped 2-D crystals up to 2×1 µm in size (Fig. 6*b, 6c*). Within these vesicles, regular crystalline patches of approximately 300×300 nm were found and they were further analyzed using 2D crystal software (see Materials and Methods).

The calculated power spectrum of negatively stained 2-D crystal images showed clear diffraction spots up to 21 Å resolution before image unbending (Fig. 6*d*). Comparison of phase residuals with the ALLSPACE program revealed p12_1_-b symmetry and a symmetrized projection map was calculated at 20 Å resolution (Fig. 6*f*, see also Table S1 and Table S2). The unit cell parameters were a = 122 Å, b = 236 Å and gamma = 90° with one unit cell containing two different entities of ∼110 Å×110 Å related by a screw axis along the b axis (e.g. one entity is outlined by a light grey square in Fig. 6*e*). The surface of each entity is significantly larger than the surface of the homodimeric bacterial half transporters MsbA or Sav1886 in post hydrolytic conformation of 75 Å×50 Å at the level of the NBDs. Moreover, this is close to the dimensions of 11×11 nm reported for the dimer of the yeast full transporter Pdr5, i.e. made of 4 NBDs [39]. Thus we propose that each entity of 110 Å×110 Å is made of at least a dimer of BmrC/D heterodimers.

## Discussion

In this study, we devise an efficient protocol to purify in high yield, and in a stable and monodisperse state, a new heterodimeric multidrug ABC transporter called BmrC/BmrD. This is a prerequisite step for biochemical and structural characterization especially for crystallization attempts [36]. For heterodimers or transporters with several subunits, if only one subunit is tagged the purification protocol must maintain the tight interactions between all components of the complex throughout the procedure, notably during the solubilization step where high concentrations of detergent are usually required [40], [41]. Clearly, the interaction observed here between BmrC/BmrD was not tight enough to efficiently co-purify BmrC with a hexahistidine-tagged BmrD in a 1/1 complex; the introduction of a second tag was therefore required to overcome this hurdle. Such a problem was apparently not encountered for one of the best-characterized heterodimeric half-size exporters, TAP1/TAP2, which could be purified as a stable complex when only one subunit was tagged [42], [43]. The BmrC and BmrD subunits each have 6 predicted transmembrane helices (Figure S5) whereas TAP1 and TAP2 have 10 and 9 predicted transmembrane helices, respectively. Possibly, the supernumerary helices in TAP1/TAP2 might confer additional stability to the transporter after detergent solubilization [4], [44], [45]. Affinity-purification of other ABC heterodimers, such as LmrC/LmrD [24], AbcA/AbcB [25] and native ABCG5/ABCG8 [46], has been reported upon tagging of a single subunit or using an antibody against only one subunit. However, the homogeneity of these preparations was not assessed, e.g. by gel filtration or analytical ultracentrifugation. Importantly, Xie and colleagues reported that ABCG5/ABCG8 heterodimers were disrupted on ion exchange or hydroxyapatite columns [46]. Similar to the strategy used here, purification of recombinant ABCG5/ABCG8 heterodimers was performed after tagging each subunit [47].

While the expression levels of BmrC and BmrD in the *E. coli* membrane were very high for the longest induction time used here, the extraction efficiency was found to be surprisingly low for many ‘mild’ detergents commonly used in membrane protein purification. Such a huge overexpression of BmrC/BmrD appeared actually detrimental to the solubilization step, because a shortening of the induction time led to much higher solubilization yields, up to 50%. Thus, the poor solubilization yield obtained (e.g. with DDM) when the highest overexpression was reached might actually reflect the selective extraction of the population of BmrC/BmrD heterodimers in a correctly folded conformation. This is in line with the poor solubilization efficacy observed for each subunit alone (Fig. S3) suggesting that in the membrane fraction and in the absence of their twin subunit, BmrC or BmrD are not properly folded and so refractory to solubilization by mild detergent. In contrast, the fos-cholines solubilize almost all protein in the membrane and destabilize the BmrC/BmrD heterodimers to a higher extent than DDM, as suggested by the important loss of untagged subunits during affinity chromatography (Fig. S1). This explains the variability in apparent BmrC/BmrD stoichiometry and specific ATPase-activity upon purification in presence of different detergents.

DDM-purified BmrC/BmrD heterodimers seem homogeneous when analyzed by gel filtration or analytical ultracentrifugation. Importantly, almost no monomeric species were detected in the analytical ultracentrifugation experiments, indicating that the destabilization of the BmrC/BmrD heterodimer during the initial solubilization step is reversible when the detergent concentration is lowered. Although we cannot rule out that some homodimers were present in our preparation, this appears rather unlikely given that (i) the propensity of our samples to readily crystallize in two dimensions and (ii) the poor yield of single subunit purification suggesting an instability of homodimers. In addition, under similar solubilization conditions (i.e. 1% DDM) the homodimeric multidrug bacterial ABC transporter BmrA was in equilibrium between monomers and dimers [18], and thus behaved as the BmrC/BmrD heterodimer. However, once the DDM concentration was lowered from 1% to 0.05% on the subsequent affinity column, BmrA reformed stable homodimers [13], [21].

The purified BmrC/BmrD heterodimer has a significant ATPase activity in detergent (∼200 nmol·min^−1^·mg^−1^), sensitive to vanadate inhibition and drug activation, which can be highly increased upon reconstitution into proteoliposomes. A remarkable feature of BmrC/BmrD ATPase activity, which highlights the purification quality, is its long-term stability in detergent. This contrasts for instance with Pgp or MRP3 which were shown to require the addition of lipids to be stable under similar conditions [48], [49]. Variable effects on the ATPase activity of different ABC exporters have been reported following reconstitution into proteoliposomes, with a marked stimulation for BmrA [13], a modest stimulation for Pgp, MRP3 or TAP1/TAP2 [50], [51], [52] or a three fold decrease for MsbA [53]. It is noteworthy that none of the reconstitution reports cited above verified the lamellarity of the obtained liposomes as done here using cryoelectron microscopy. The presence of multilamellar liposomes in the proteoliposome preparation can substantially diminish the ATPase activity, as observed here for OTG-mediated reconstitutions (Fig. S4), and could partially explain the large variability of reconstitution previously reported for different ABC transporters. Furthermore, the high ATPase activity measured here for BmrC/BmrD is specific because mutation of the invariant lysine residue to an alanine in the Walker-A motifs of both BmrC and BmrD led to an affinity-purified mutant transporter with a low residual ATPase activity in detergent (cf. Fig. S2 *b*). This activity became virtually undetectable after gel filtration and upon reconstitution into proteoliposomes (not shown).

Either in a detergent solubilized state or reconstituted in proteoliposomes, BmrC/BmrD ATPase activity is stimulated by its Hoechst 33342 substrate. This observation suggests that the global conformation of this heterodimer and its coupling mechanism is conserved in the DDM-solubilized state. Yet, maximal stimulation of the reconstituted transporter was achieved at lower drug concentrations, suggesting that its substrate affinity and/or coupling mechanism between transmembrane and nucleotide-binding domains were favored upon reconstitution. While we previously showed by using inside out vesicles that the multidrug BmrC/BmrD transporter catalyzed the transport of several drugs [31], not every substrate was found here to stimulate the ATPase activity of the transporter. This observation is in agreement with previous reports on other ABC transporters, e.g. MsbA [53], Cdr1 [54] or ABCC3 [49]. Different drugs can interact differently with the same ABC transporter, both structurally (having different binding sites [55]) and thermodynamically (causing different interactions with the rate-limiting transition state [56]). Thus different effects ranging from stimulating to inhibitory can be elicited by different substrates on the ATPase activity of their cognate transporter.

Despite the apparent efficiency of the reconstitution protocol used in this work and the conservation of the coupling mechanism in the purified BmrC/BmrD transporter, we have been unable to detect a reliable drug transport activity in our proteoliposome preparations. Although there is currently no satisfactory explanation for this, it is noteworthy that a successful reconstitution protocol to monitor peptide transport using highly purified TAP1/TAP2 took several years to be achieved, and this exemplifies the intrinsic difficulty when one works with membrane proteins [42], [51], [57]. Possibly, an additional component present in the *E. coli* membrane vesicles but missing in our reconstitution protocol might be required. Very recently, HorA was shown to lose its transport activity when PE was replaced with PC in the lipid mixture used for reconstitution [58] and purified LmrC/LmrD was shown to contain one or two molecules of cardiolipin associated with it [59]. Addition of cardiolipin and varying the ratio and nature of commonly used lipids did not allow us to monitor some transport with reconstituted BmrC/BmrD, and whether some specific or unusual lipids are necessary for the drug transport activity of BmrC/BmrD will need further investigation.

The quaternary structure of BmrC/BmrD is a heterodimer in detergent solution, but can be assembled into a higher oligomeric form in a lipid membrane environment. The quaternary structure of ABC transporters is a highly debated question, since higher oligomeric states than the minimal functional unit (i.e. two TMDs plus two NBDs) have been reported for various ABC transporters, including CFTR [60], [61], [62], [63], BCRP [64], [65], [66], [67] or ABCA1 [68] (for a recent review see [69]). For example, MRP1 contains two asymmetrical NBDs, one of which with non canonical residues like those in BmrC [31], and was reported to be monomeric in detergent solution and dimeric after reconstitution into a lipid membrane [70]. Interestingly, the oligomeric state has been proposed to regulate the function of some ABC transporters, e.g. ABCA1 [68] or BCRP [66], [71]. Phosphorylation of the latter by the Pim-1L protein kinase promotes its multimerization and modulates the BCRP-mediated drug resistance phenotype in prostate cancer cells [66]. However, X-ray structures available for ABC exporters (full-length murine P-glycoprotein and half-size homodimeric ABC transporters, MsbA or Sav1886), have been solved in a detergent solution. In this context, 2D crystallization can provide information, although at lower resolution, on protein assembly in a lipidic environment and on conformational changes triggered by the binding of effectors. For instance, the shape of membranes and 2D crystals of BmrA were modified upon ATP binding [72]. For membrane bound MsbA, helical arrays of the transporter were obtained and their analysis by Ward and colleagues showed that MsbA remains in an outward facing conformation when trapped in three different transition states using different combinations of nucleotides and inhibitors [73]. For human P-glycoprotein, a central cavity was observed in the nucleotide free state, as opposed to vanadate trapped states, and intermediate conformations were also detected in the presence of ADP alone [74]. Here, crystals of BmrC/BmrD were obtained only in an ADP/Vi trapped conformation, and the projection map calculated at 20 Å resolution showed two entities of ∼110 Å×110 Å that could accommodate four NBDs. This highlights the possibility that BmrC/BmrD associates into dimers of heterodimers in the membrane. Further experiments are currently in progress to improve the quality of the 2D crystals and to obtain a more detailed description of protein/protein interactions in the membrane. Yet, given the paucity of structural data for asymmetric transporters in this conformation and in a lipid membrane, this work represents an important contribution to the structural understanding of ABC transporters in general.

## Materials and Methods

### Protein overexpression

*yheI (bmrC)* and *yheH (bmrD)* genes were amplified from *Bacillus subtilis 168* genome and cloned into a modified pET21b(+) vector for introducing one His_6_-tag at the N-terminus of BmrC and another one at the C-terminus of BmrD. Protein overexpression in *Escherichia coli* BL21(DE3) and isolation of the membrane fraction enriched with BmrC and BmrD proteins were performed as described [31], except that the induction time was reduced to 6 hours.

### Detergent screen and protein purification

The resulting membrane fraction was solubilized at a detergent/protein ratio of 5–10 (w/w) in buffer A (50 mM Tris-HCl pH 8, 100 mM NaCl, 15% (v/v) glycerol, 5 mM ß-mercaptoethanol, 10 mM imidazole) with the following detergents: DDM, DOTM, OG, HECAMEG, LDAO, FC-12, FC-16, Z3-12 and CHAPS. After incubation for 2–3 hours at 4°C, soluble and insoluble fractions were separated by centrifugation at 180 000 g for 60 min and analyzed by SDS-PAGE. The soluble fraction was then incubated with 1/10 (v/v) of Ni^2+^-IDA resin (*Prochem*) for 1 h. The resin was washed with 25 column volumes of buffer A′ (buffer A supplemented with 20 mM imidazole and 0.005% (w/v) FC-16, 0.05% (w/v) DDM, 0.05% (w/v) LDAO or 0.1% (w/v) FC-12). Elution was done in the same buffer but in the presence of 250 mM imidazole. Purified proteins were dialysed against 50 mM Tris-HCl pH 8, 50 mM NaCl, 10% (v/v) glycerol, 5 mM ß-mercaptoethanol, in the presence of the same detergent and at the same concentration as the elution buffer. All steps were carried out at 4°C or on ice. Purified proteins were frozen in liquid N_2_ and stored at −80°C. Protein concentration was determined using the Bradford assay (*Coomassie Plus, Pierce*). Size-exclusion chromatography (SEC) of 0.1–1 mg affinity-purified proteins was performed on a Superdex 200 column (10/300 GL, *GE Healthcare*), using buffer B (50 mM Tris-HCl pH 8, 150 mM NaCl, 10% (v/v) glycerol, 5 mM ß-mercaptoethanol, 0.05% (w/v) DDM) as the running buffer.

### Analytical ultracentrifugation

Samples from the affinity chromatography were used either directly or submitted to an additional SEC step. Major peak fractions from SEC experiments were pooled and the BmrC/BmrD proteins were kept at 4°C for the indicated times before sedimentation-velocity experiments were run. Analytical ultracentrifugation experiments were performed on a XLI Beckman ultracentrifuge, at 20°C and 42000 rpm and analyzed as described previously [75], [76]. The reference solvent used was without detergent. From the amino-acid composition of BmrC/BmrD, a molar mass and partial specific volume of 144.97 kDa and 0.745 ml/g were calculated and a refractive index increment of 0.187 g/ml was used. For DDM a partial specific volume of 0.82 ml/g and a refractive index increment of 0.143 g/ml were used [77]. A solvent density of 1.026 g/ml and a viscosity of 1.33 cP were measured at 20°C. The *c*(*s*) analysis [78] and conversion of the experimental values of the sedimentation coefficients, *s*_exp_, to corrected values, *s*_20w_, were done using a partial specific volume for the protein-DDM complex of 0.78 ml/g.

### Reconstitution of BmrC/BmrD

Proteoliposomes were prepared from a ternary protein/lipid/detergent mixture by adsorption of detergent onto polystyrene beads (Bio-Beads SM-2, Biorad) according to [79]. Different lipids as indicated (from *Avanti Polar Lipids*), stored in chloroform, were mixed and evaporated under N_2_ gas. After complete evaporation of chloroform under vacuum, the lipidic film was solubilized for 16 h at a detergent/lipid ratio of 2–3 (w/w) and a lipid concentration of 4 mg/ml, in 50 mM Tris-HCl pH 8, 150 mM KCl, 25 mM NaCl, 4% (v/v) glycerol, 1 mM ß-mercaptoethanol. The BmrC/BmrD proteins were incubated for 15 min with the lipid/detergent mixture at a lipid/protein ratio of 40 (w/w), and 3 successive additions of Bio-Beads (first followed by a 2-h incubation, then twice by a 1-h incubation) were performed at a Bio-Beads/detergent ratio of 20 (w/w). For direct incorporation, preformed liposomes of 4 mg/ml PC/PA (9/1 w/w) were treated with 2.4 mg/ml DDM leading to the saturation of membrane with DDM [80]. Protein was added at a lipid to protein ratio of 40 (w/w). The detergent was removed using Bio-Beads as described above. All steps were carried out at room temperature. Proteoliposomes were kept at 4°C and activity measurements were measured within 48 h.

The incorporation of BmrC/BmrD in liposomes was measured after reconstitution at a lipid/protein of 40 (w/w) from fully solubilized PC/PA lipids. Proteoliposomes were submitted to discontinuous flotation gradients as previously described with successive layers containing 500 µl of 30, 20, 10, 5 and 2.5% sucrose concentration (w/w) using a TLA 100 centrifuge tube (11×54 mm) [37]. After 16-hour centrifugation at 4°C and 25000 rpm in a TLA 100 rotor (24115×g), the fractions were collected and 10 µl of each fraction was analyzed for protein contents by immunoblot using HisProbe-HRP (Thermo Scientific). The intensities of the bands corresponding to BmrC/BmrD were quantitated using Image Gauge software (Fuji Film Science Lab) and normalized to the total amount of BmrC/BmrD in the gradient.

### ATPase activity

The ATPase activity of BmrC/BmrD was measured using an enzymatic assay that allows ATP regeneration to be coupled to NADH oxidation, which is recorded at 340 nm. ATPase activity of BmrC/BmrD in detergent (10 µg) was measured at 30°C in buffer B supplemented with 32 µg/ml lactate dehydrogenase (*Roche*), 60 µg/ml pyruvate kinase (*Roche*), 4 mM phosphoenolpyruvate, and 0.4 mM NADH. For the ATPase activity of reconstituted BmrC/BmrD (3 µg), buffer B was replaced by 50 mM Hepes pH 8 and measurements were done at 37°C. Unless stated otherwise, ATP and MgCl_2_ were added at 4 mM and 5 mM, respectively. Hoechst 33342 (Sigma) was dissolved at 1 mM in deionized water, and ortho-vanadate (Sigma) was prepared at 100 mM as described previously [81] and heated at 95°C for 5 min before use. The initial rate of ATP hydrolysis was plotted against the ATP concentration, and curve fitting was performed using GraFit 5.0 (Erithacus Software). To avoid any interference with the enzymes in the coupled enzymatic assay, ATPase activity in the presence of fos-choline or LDAO was measured by a colorimetric assay of Pi release, as previously described [82].

### 2-D crystallisation and electron microscopy

50 µl of BmrC/BmrD at 0.5 mg/ml solubilized in 0.1% DOTM was supplemented with a solubilized mixture of EPC/EPA/cholesterol 7/1/2 (mol/mol) at lipid/protein ratio of 0.6–0.8 (w/w) in 50 mM Tris-HCl pH 8, 200 mM NaCl, 2.5 mM MgCl_2_ supplemented with 5 mM ATP and 2 mM ortho-vanadate. To ensure a complete mixing of lipids and detergent, solubilized lipids were prepared by mixing lipids and DOTM 1/4 (w/w) in chloroform and after its evaporation, the dried film was resuspended in water leading to a clear suspension. After overnight incubation at 4°C of lipids, proteins and detergent, detergent was slowly removed by 3 additions of 1 mg of Bio-Beads without stirring, each addition being followed by a 24 hours incubation period [83]. Vesicles appeared the second day while 2-D crystals were observed the third day. 2-D crystals were stable for a week at 4°C.

For electron microscopy, 2-D crystals were negatively stained with 2% uranyl acetate. Images were recorded under low dose conditions on a SSC GATAN camera 1024×1024 pixel with a CM120 Phillips electron microscope operating at 120 kV. Images were recorded at a magnification of 45000 corresponding to a resolution of 3.86 Å/pixel. Image analysis was performed with 2dx package which included two cycle of lattice unbending (CCUNBENDK), calculation of defocus and astigmatism values (CTFFIND) and correction of the contrast Fourier transform (CTFAPPLY). Plane symmetry was established by analysis of the images using ALL SPACE (Valpuesta et al., 1994) with IQ 1–5 up to 20 Å. The five best images ranging in defocus from −0.4 to −0.8 µm were merged and used to calculate a projection map at 20 Å resolution with p12_1_-b symmetry (see Table S1 and Table S2).

For cryo-electron microscopy of proteoliposomes, samples were flash frozen in liquid ethane and images were recorded under low dose conditions.

## Supporting Information

Figure S1 **Purification of BmrC/BmrD.** Left panel, only the BmrD subunit is histidine-tagged. Right panel, both BmrC and BmrD are histidine-tagged. Lanes 1 and 5, membrane fractions; lanes 2 and 6, supernatants obtained after membrane solubilization with 1% FC12 (lanes 2) or 1% DDM (lanes 6) and centrifugation (180,000 g, 1 h); lanes 3 and 7, unbound material not retained on the Ni-Agarose chromatography column; lanes 4 and 8, material eluted from the Ni-Agarose chromatography column in the presence of 250 mM imidazole. Positions of molecular weights markers are indicated (in kDa) and those of BmrC and BmrD are shown by grey or black arrowheads, respectively. Please note that for the mono-tag construct (left panel), more BmrC subunit is lost in the ‘unbound’ fraction (lanes 4 and 7) resulting in a non-stoichiometric recovery of BmrC/BmrD.
(PDF)

Figure S2 **ATPase activity of BmrC/BmrD purified using different detergents.** The ATPase activity was measured at 4 mM ATP and the value obtained with DDM (140 nmol·min^−1^·mg^−1^) was taken as 100%.
(PDF)

Figure S3 **Purification of BmrC or BmrD proteins overexpressed alone and ATPase activities.** ***(a)*** lanes 1, membrane fractions; lanes 2, supernatants obtained after solubilization with 1% DDM; lanes 3, unbound materials not retained on the Ni-Agarose chromatography columns; lanes 4, proteins eluted from the Ni-Agarose chromatography column in the presence of 250 mM imidazole. Positions of molecular weight markers are indicated (in kDa). ***(b)*** ATPase activities of BmrC and BmrD purified separately (cf. lanes 4 in ***a***) are compared to that displayed by both proteins after their co-expression and joint purification (either the wild-type transporter, BmrC/BmrD, or a double mutant BmrC K377A/BmrD K469A). Measurements were performed with 0.05% DDM as described in the Experimental Procedures section, in the presence (+) or absence (−) of 0.5 mM vanadate. Representative values are shown, expressed as the mean ± SD for three measurements for experiments performed in the absence of vanadate.
(PDF)

Figure S4 **ATPase activity of BmrC/BmrD proteoliposomes prepared in the presence of different detergents.** PC/PA (9∶1 molar ratio) were solubilized with the indicated detergent prior to incubation with purified BmrC/BmrD, and detergent was removed using BioBeads. DM, decylmaltoside; TX-100, Triton X-100; OTG, octylthioglucoside. The obtained proteoliposomes were imaged using Cryo-TEM (*b–d*), and their ATPase activity was assessed at 4 mM ATP (*a*). ATPase activities are expressed relative to the value obtained for BmrC/BmrD reconstituted into DM solubilized PC/PA (1.9±0.4 µmol·min^−1^·mg^−1^; n = 5).
(PDF)

Figure S5 **Alignment of BmrC (YheI) and BmrD (YheH) proteins.** Protein sequences were obtained from the Subtilist Web Server (http://genolist.pasteur.fr/SubtiList/). The alignment was generated by ClustalW (1.8) on the NPS@ server (http://npsa-pbil.ibcp.fr) using the default parameters except that PAM was used as the weight matrix in the multiple alignment parameters window. The figure was made with ESPript 2.0 (http://prodes.toulouse.inra.fr/ESPript/). A blue frame was drawn when two residues were similar (red letters in yellow boxes) or identical (white letters in red boxes). The approximate boundary between the transmembrane domain (TMD) and nucleotide-binding domain (NBD) is indicated. For the NBD, the Walker A (WA) and B (WB) motifs and the ABC signature (ABC) are underlined. For the TMD, the location of the putative transmembrane helices (numbered TMH1 to 6) is indicated above or below the sequence for BmrC and BmrD, respectively, using the prediction obtained from the following web servers: TopPred (http://mobyle.pasteur.fr/cgi-bin/portal.py?form=toppred; cyan colored), TMHMM v. 2.0 (http://www.cbs.dtu.dk/services/TMHMM/; yellow-green colored), PHDhtm (http://npsa-pbil.ibcp.fr/cgi-bin/npsa_automat.pl?page=/NPSA/npsa_htm.html; teal colored), HMMTOP (http://www.enzim.hu/hmmtop/index.html; dark blue colored) and DAS (http://www.sbc.su.se/~miklos/DAS/maindas.html; green colored). For TMHMM, PHDhtm, HMMTOP and DAS programs, the default parameters were used. For TMHMM, this allowed the prediction of 6 and 5 transmembrane helices for BmrC and BmrD, respectively; the fifth putative transmembrane helix of BmrD was not predicted to be long enough to give rise to two different transmembrane helices. With the PHDhtm program, only 5 transmembrane helices were predicted for both BmrC and BmrD proteins, but the third one is very long in both cases and thus is possibly split into two shorter transmembrane helices (the same explanation holds true for the transmembrane predicition of BmrD with the DAS program). With the latter program, only transmembrane helices whose maximal prediction values were above the strict cutoff (>2.2) were considered. For BmrC, the DAS program identified an additional putative transmembrane helice that was, however, only 8 residues long (133–140), and thus is not shown in the figure (this stretch likely represents the beginning of TMH3 of BmrC which is otherwise predicted to start at residue 143). With the TopPred program, the default parameters used initially predicted only 5 and 4 transmembrane helices for BmrC and BmrD, respectively. However, the use of less stringent parameters, as indicated below, with the hydrophobicity GES-scale allowed the prediction of 6 transmembrane helices for both proteins as shown in the figure; full window size: 18 (BmrC) and 14 (BmrD); core window size: 8 (BmrC) and 6 (BmrD); wedge window size: 5 (BmrC) and 4 (BmrD); cutoff for certain and putative transmembrane segments: 1.4 and 1.1, respectively; critical distance between 2 transmembrane segments: 1 and Critical loop length: 1.
(PDF)

Table S1 **Phase Residuals in Resolution Ranges.** Phase residuals in degrees, obtained during merging of five negatively stained 2D crystal image data. Columns show resolution range in Å. IQ = 1….8, intensity quotient categories of reflections (1); All IQs, average phase residuals with equal reflection weighting; IQ-wght, average phase residual with IQ-weighting. In each resolution range (Dmin to Dmax in Å), the phase residuals (upper row) and the number of reflections (lower row) are given. This table gives suitable phase residuals up to 20 Å resolution and the projection map data were limited to this resolution. Reference: (1) Henderson, R., Baldwin, J.M., Ceska, T.A., Zemlin, F., Beckmann, E. and Downing, K.H. (1990). *J. Mol. Biol.* **213**, 899–929.
(PDF)

Table S2 **Internal phase residuals of all possible two-sided plane groups using a representative image.** Internal phase residuals were determined using the program ALLSPACE (Valpuesta *et al.*, 1994) from spots of IQ1 to IQ5 to 20 Å resolution. ^a^ Phase residual *versus* other spots (90° random). ^b^ Target residual based on statistics taking Friedel weight into account. ^c^ Note that in space group *p*1 no phase comparison is possible, so the numbers given here are theoretical phase residuals based on the signal-to-noise ratio of the observed diffraction spots. * acceptable, ! should be considered, ‘possibility.
(PDF)
