# Supplementary figures and images for: Optimized Purification of a Heterodimeric ABC Transporter in a Highly Stable Form Amenable to 2-D Crystallization
Source: PLoS One. 2011 May 13;6(5):e19677. doi: 10.1371/journal.pone.0019677 (PMC3094339; doi:10.1371/journal.pone.0019677)

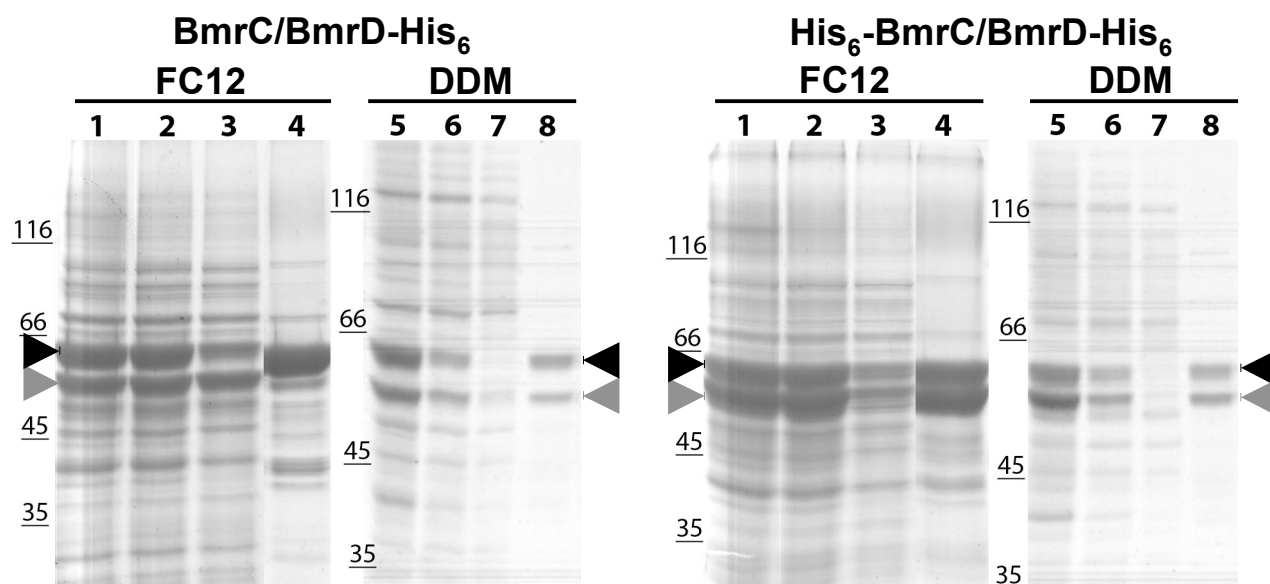

**Figure S1**

Supplement: Figure S1 — Purification of BmrC/BmrD. Left panel, only the BmrD subunit is histidine-tagged. Right panel, both BmrC and BmrD are histidine-tagged. Lanes 1 and 5, membrane fractions; lanes 2 and 6, supernatants obtained after membrane solubilization with 1% FC12 (lanes 2) or 1% DDM (lanes 6) and centrifugation (180,000 g, 1 h); lanes 3 and 7, unbound material not retained on the Ni-Agarose chromatography column; lanes 4 and 8, material eluted from the Ni-Agarose chromatography column in the presence of 250 mM imidazole. Positions of molecular weights markers are indicated (in kDa) and those of BmrC and BmrD are shown by grey or black arrowheads, respectively. Please note that for the mono-tag construct (left panel), more BmrC subunit is lost in the ‘unbound’ fraction (lanes 4 and 7) resulting in a non-stoichiometric recovery of BmrC/BmrD. (PDF) [file pone.0019677.s001.pdf]

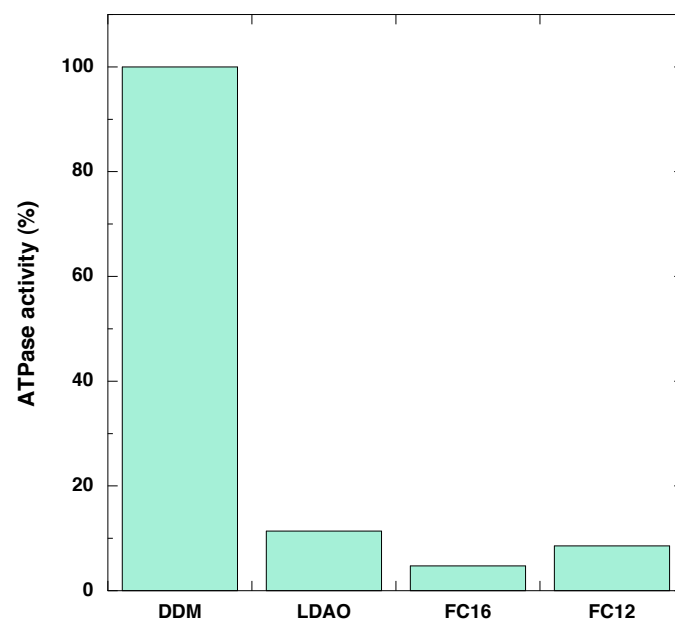

**Figure S2**

Supplement: Figure S2 — ATPase activity of BmrC/BmrD purified using different detergents. The ATPase activity was measured at 4 mM ATP and the value obtained with DDM (140 nmol·min−1·mg−1) was taken as 100%. (PDF) [file pone.0019677.s002.pdf]

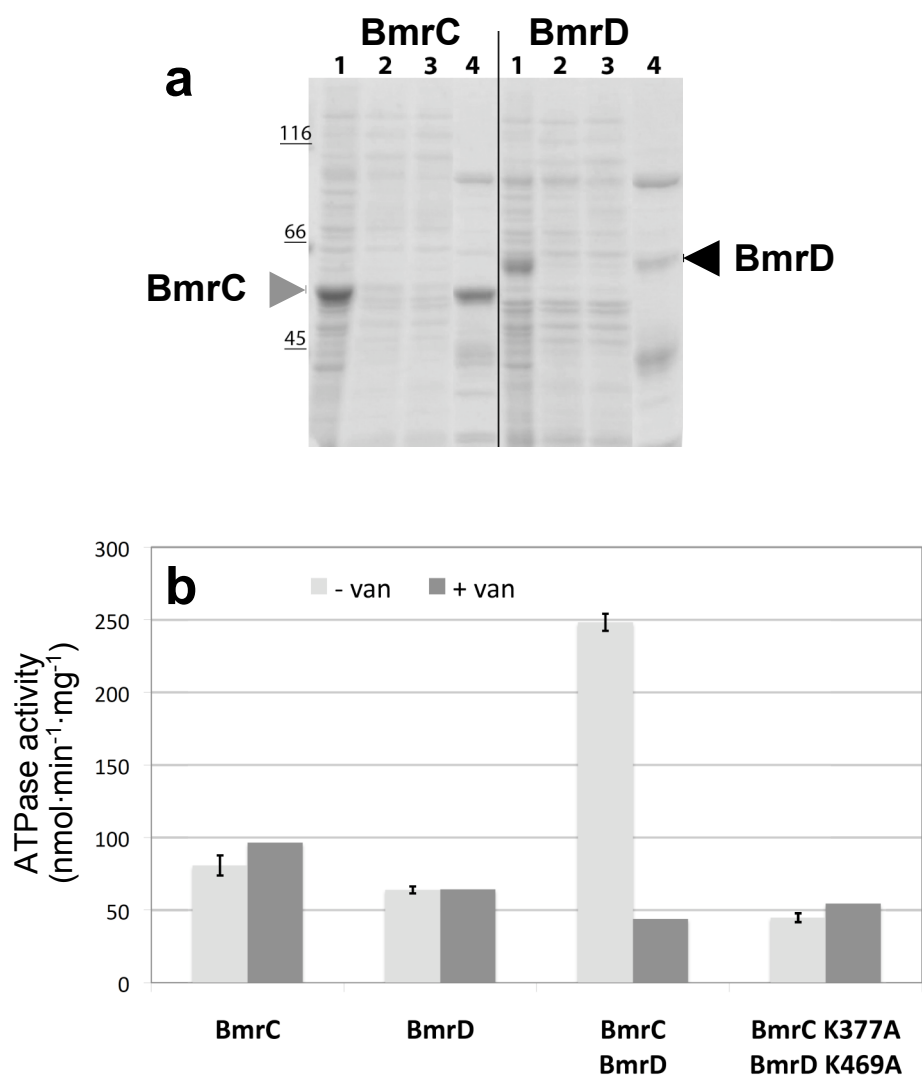

**Figure S3**

Supplement: Figure S3 — Purification of BmrC or BmrD proteins overexpressed alone and ATPase activities. (a) lanes 1, membrane fractions; lanes 2, supernatants obtained after solubilization with 1% DDM; lanes 3, unbound materials not retained on the Ni-Agarose chromatography columns; lanes 4, proteins eluted from the Ni-Agarose chromatography column in the presence of 250 mM imidazole. Positions of molecular weight markers are indicated (in kDa). (b) ATPase activities of BmrC and BmrD purified separately (cf. lanes 4 in a) are compared to that displayed by both proteins after their co-expression and joint purification (either the wild-type transporter, BmrC/BmrD, or a double mutant BmrC K377A/BmrD K469A). Measurements were performed with 0.05% DDM as described in the Experimental Procedures section, in the presence (+) or absence (−) of 0.5 mM vanadate. Representative values are shown, expressed as the mean ± SD for three measurements for experiments performed in the absence of vanadate. (PDF) [file pone.0019677.s003.pdf]

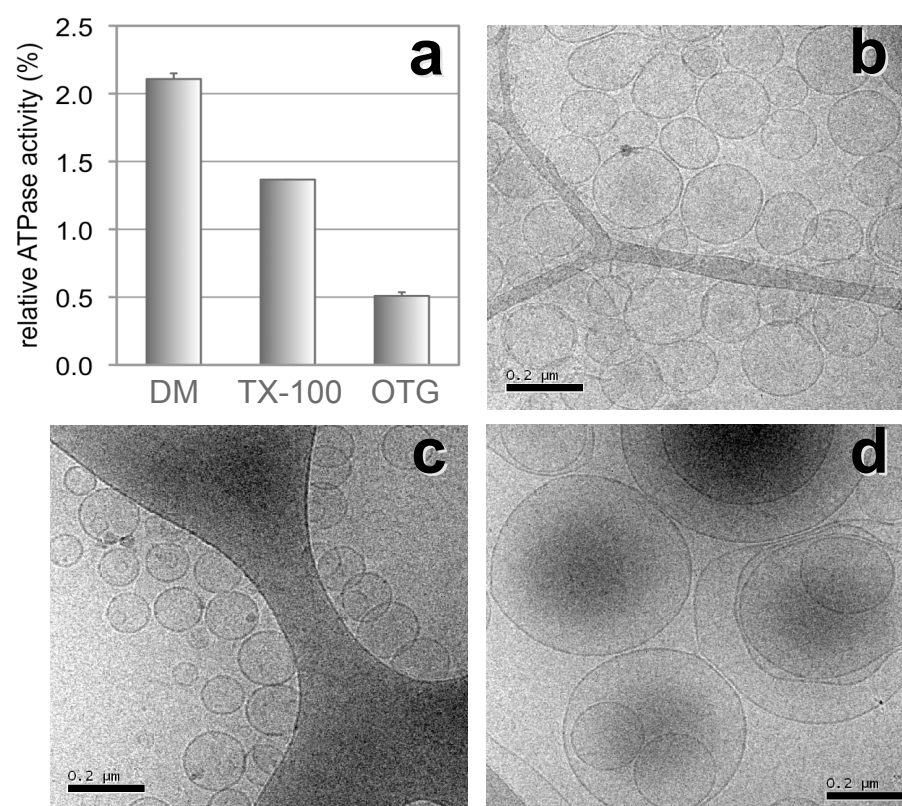

**Figure S4**

Supplement: Figure S4 — ATPase activity of BmrC/BmrD proteoliposomes prepared in the presence of different detergents. PC/PA (9∶1 molar ratio) were solubilized with the indicated detergent prior to incubation with purified BmrC/BmrD, and detergent was removed using BioBeads. DM, decylmaltoside; TX-100, Triton X-100; OTG, octylthioglucoside. The obtained proteoliposomes were imaged using Cryo-TEM (b–d), and their ATPase activity was assessed at 4 mM ATP (a). ATPase activities are expressed relative to the value obtained for BmrC/BmrD reconstituted into DM solubilized PC/PA (1.9±0.4 µmol·min−1·mg−1; n = 5). (PDF) [file pone.0019677.s004.pdf]

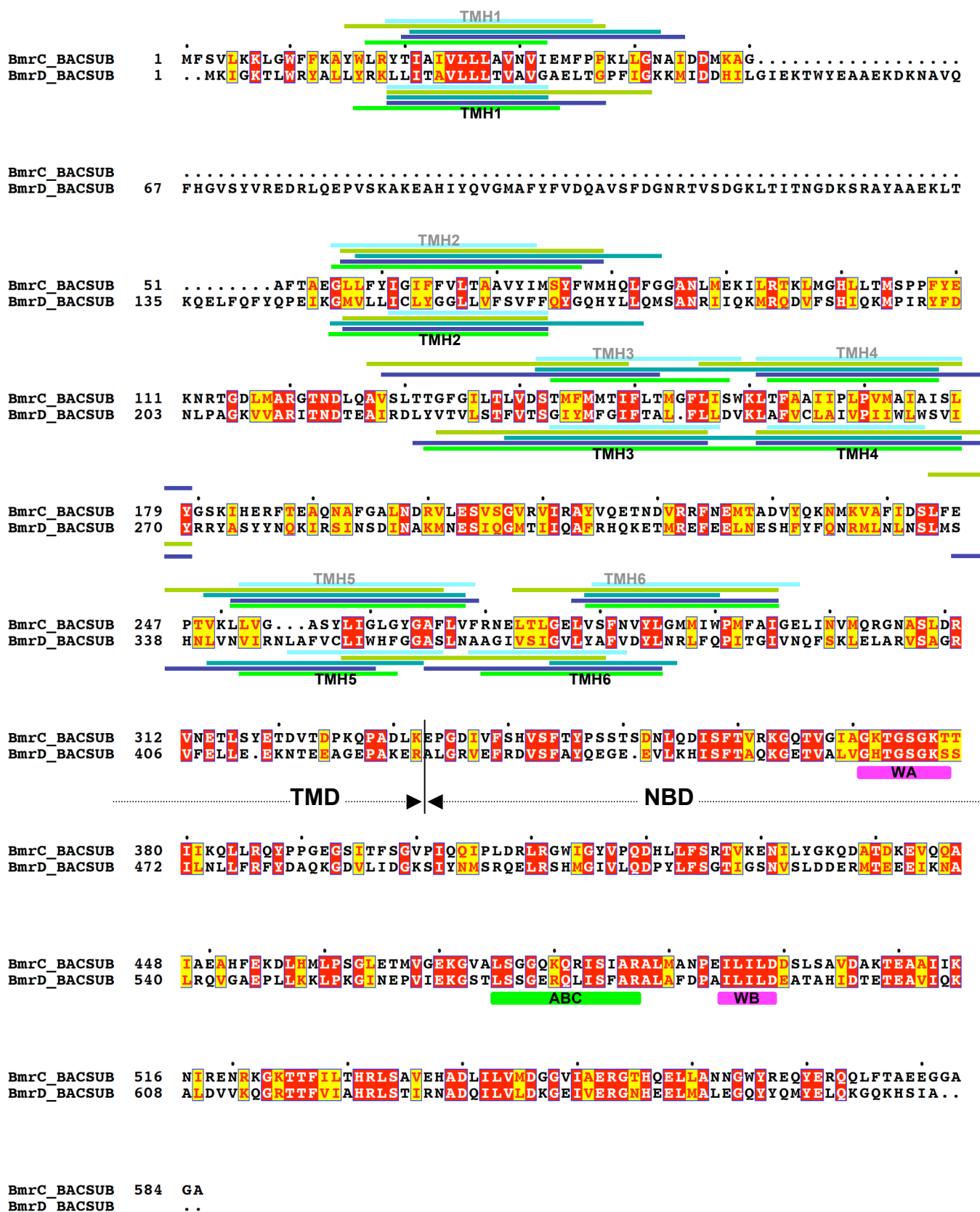

Figure S5

Supplement: Figure S5 — Alignment of BmrC (YheI) and BmrD (YheH) proteins. Protein sequences were obtained from the Subtilist Web Server (http://genolist.pasteur.fr/SubtiList/). The alignment was generated by ClustalW (1.8) on the NPS@ server (http://npsa-pbil.ibcp.fr) using the default parameters except that PAM was used as the weight matrix in the multiple alignment parameters window. The figure was made with ESPript 2.0 (http://prodes.toulouse.inra.fr/ESPript/). A blue frame was drawn when two residues were similar (red letters in yellow boxes) or identical (white letters in red boxes). The approximate boundary between the transmembrane domain (TMD) and nucleotide-binding domain (NBD) is indicated. For the NBD, the Walker A (WA) and B (WB) motifs and the ABC signature (ABC) are underlined. For the TMD, the location of the putative transmembrane helices (numbered TMH1 to 6) is indicated above or below the sequence for BmrC and BmrD, respectively, using the prediction obtained from the following web servers: TopPred (http://mobyle.pasteur.fr/cgi-bin/portal.py?form=toppred; cyan colored), TMHMM v. 2.0 (http://www.cbs.dtu.dk/services/TMHMM/; yellow-green colored), PHDhtm (http://npsa-pbil.ibcp.fr/cgi-bin/npsa_automat.pl?page=/NPSA/npsa_htm.html; teal colored), HMMTOP (http://www.enzim.hu/hmmtop/index.html; dark blue colored) and DAS (http://www.sbc.su.se/~miklos/DAS/maindas.html; green colored). For TMHMM, PHDhtm, HMMTOP and DAS programs, the default parameters were used. For TMHMM, this allowed the prediction of 6 and 5 transmembrane helices for BmrC and BmrD, respectively; the fifth putative transmembrane helix of BmrD was not predicted to be long enough to give rise to two different transmembrane helices. With the PHDhtm program, only 5 transmembrane helices were predicted for both BmrC and BmrD proteins, but the third one is very long in both cases and thus is possibly split into two shorter transmembrane helices (the same explanation holds true for the transmembrane predicition of BmrD [file pone.0019677.s005.pdf]
