# Supplementary material for: Optimized Purification of a Heterodimeric ABC Transporter in a Highly Stable Form Amenable to 2-D Crystallization
Source: PLoS One. 2011 May 13;6(5):e19677. doi: 10.1371/journal.pone.0019677 (PMC3094339; doi:10.1371/journal.pone.0019677)
Supplement: Table S1 — Phase Residuals in Resolution Ranges. Phase residuals in degrees, obtained during merging of five negatively stained 2D crystal image data. Columns show resolution range in Å. IQ = 1….8, intensity quotient categories of reflections (1); All IQs, average phase residuals with equal reflection weighting; IQ-wght, average phase residual with IQ-weighting. In each resolution range (Dmin to Dmax in Å), the phase residuals (upper row) and the number of reflections (lower row) are given. This table gives suitable phase residuals up to 20 Å resolution and the projection map data were limited to this resolution. Reference: (1) Henderson, R., Baldwin, J.M., Ceska, T.A., Zemlin, F., Beckmann, E. and Downing, K.H. (1990). J. Mol. Biol. 213, 899–929. (PDF) [file pone.0019677.s006.pdf]

PHASE RESIDUALS IN RESOLUTION RANGES

#	DMIN	DMAX	IQ=1	2	3	4	5	6	7	8 all	IQs	IQ-wght
1	1000.0	73.7	7.1	14.6	0.0	0.0	0.0	112.9	156.2	96.8	34.2	14.3
1	16	4	0	0	0	1	2	3	26	26		
2	73.5	52.1	4.5	21.7	70.2	93.0	160.4	81.5	0.0	104.0	35.0	22.4
2	16	11	3	3	1	2	0	2	38	38		
3	52.1	42.6	10.5	45.1	47.7	21.8	12.4	21.6	0.0	0.0	29.3	29.9
3	4	3	5	2	2	2	0	0	18	18		
4	42.5	36.9	6.0	4.4	29.7	18.8	15.6	37.7	9.9	78.9	14.7	8.4
4	22	10	5	1	2	1	1	3	45	45		
5	36.8	33.0	3.9	11.7	10.4	13.4	0.0	42.5	0.0	0.9	11.6	11.0
5	3	13	3	3	0	1	0	1	24	24		
6	33.0	30.1	0.0	16.4	22.2	20.8	61.2	121.8	0.0	51.1	33.6	26.2
6	0	8	6	3	5	1	0	1	24	24		
7	30.1	27.9	8.8	8.3	21.2	22.6	43.4	111.2	0.0	62.2	31.5	20.6
7	1	14	4	7	6	4	0	2	38	38		
8	27.9	26.1	6.2	11.6	0.0	66.5	0.0	0.0	20.7	0.0	23.7	18.0
8	3	6	0	3	0	0	1	0	13	13		
9	26.1	24.6	0.0	0.0	0.0	25.5	18.2	23.1	20.2	54.3	38.1	22.1
9	0	0	0	1	1	3	2	7	14	14		
10	24.6	23.3	0.0	10.3	15.4	32.3	20.5	24.0	39.7	19.1	23.3	20.6
10	0	2	6	3	4	2	4	2	23	23		
11	23.3	22.2	0.0	1.8	22.0	18.2	26.3	33.6	41.0	36.1	26.7	22.2
11	0	1	4	3	5	2	2	3	20	20		
12	22.2	21.3	0.0	15.9	0.0	22.4	0.0	14.4	0.0	30.4	20.0	18.0
12	0	1	0	2	0	2	0	1	6	6		
13	21.3	20.4	0.0	0.0	0.0	17.5	0.0	21.4	0.0	52.3	35.9	19.1
13	0	0	0	1	0	1	0	2	4	4		
14	20.4	19.7	0.0	0.0	13.3	35.9	41.0	26.3	0.0	52.0	41.5	29.0
14	0	0	2	1	4	2	0	9	18	18		
15	19.7	19.0	0.0	0.0	0.0	0.0	0.0	129.2	0.0	63.7	76.8	129.2
15	0	0	0	0	0	1	0	4	5	5		
			6.1	13.5	27.7	32.6	39.3	55.3	52.0	57.4		
			65	73	38	33	30	25	12	40		

Overall: Phaseresidual = 28.462 Number of spots = 316

Table S1
