# Supplementary material for: Optimized Purification of a Heterodimeric ABC Transporter in a Highly Stable Form Amenable to 2-D Crystallization
Source: PLoS One. 2011 May 13;6(5):e19677. doi: 10.1371/journal.pone.0019677 (PMC3094339; doi:10.1371/journal.pone.0019677)
Supplement: Table S2 — Internal phase residuals of all possible two-sided plane groups using a representative image. Internal phase residuals were determined using the program ALLSPACE (Valpuesta et al., 1994) from spots of IQ1 to IQ5 to 20 Å resolution. a Phase residual versus other spots (90° random). b Target residual based on statistics taking Friedel weight into account. c Note that in space group p1 no phase comparison is possible, so the numbers given here are theoretical phase residuals based on the signal-to-noise ratio of the observed diffraction spots. * acceptable, ! should be considered, ‘possibility. (PDF) [file pone.0019677.s007.pdf]

Plane group	Phase. Residual (degree) [a]	Number. of comparison	Target residual (degree) [b]
p1	24.8 [c]	178	
p2	41.0!	89	36.2
p12_b	79.5	64	25.9
p12_a	31.6`	67	26.3
$p12_1$_b	19.0*	64	25.9
$p12_1$_a	77.2	67	26.3
c12_b	79.5	64	25.9
c12_a	31.6`	67	26.3
p222	65.2	220	29.4
$p222_1b$	64.9	220	29.4
$p222_1a$	32.1!	220	29.4
$p22_12_1$	62.5	220	29.4
c222	65.2	220	29.4
p4	72.3	185	30.3
p422	68.1	402	27.3
$p42_12$	65.6	402	27.3
p3	65.7	84	24.8
$p3_12$	69.9	213	25.3
$p32_1$	68.7	224	25.8
p6	71.2	257	28.8
p622	72.7	526	26.7

Table S2
